# Concordance of *in vitro* and *in vivo* measures of non-replicating rotavirus vaccine potency

**DOI:** 10.1016/j.vaccine.2022.07.017

**Published:** 2022-08-12

**Authors:** David McAdams, Marcus Estrada, David Holland, Jasneet Singh, Nishant Sawant, John M. Hickey, Prashant Kumar, Brian Plikaytis, Sangeeta B. Joshi, David B. Volkin, Robert Sitrin, Stan Cryz, Jessica A. White

**Affiliations:** a PATH, 2201 Westlake Ave, Seattle, WA 98122, United States; b Department of Pharmaceutical Chemistry, Vaccine Analytics and Formulation Center, University of Kansas, 2030 Becker Drive, Lawrence, KS 66047, United States; c BioStat Consulting, LLC, 10429, Big Canoe, Jasper, GA 30143-5125, United States

## Abstract

Rotavirus infections remain a leading cause of morbidity and mortality among infants residing in low- and middle-income countries. To address the large need for protection from this vaccine-preventable disease we are developing a trivalent subunit rotavirus vaccine which is currently being evaluated in a multinational Phase 3 clinical trial for prevention of serious rotavirus gastroenteritis. Currently, there are no universally accepted *in vivo* or *in vitro* models that allow for correlation of field efficacy to an immune response against serious rotavirus gastroenteritis. As a new generation of non-replicating rotavirus vaccines are developed the lack of an established model for evaluating vaccine efficacy becomes a critical issue related to how vaccine potency and stability can be assessed. Our previous publication described the development of an *in vitro* ELISA to quantify individual vaccine antigens adsorbed to an aluminum hydroxide adjuvant to address the gap in vaccine potency methods for this non-replicating rotavirus vaccine candidate. In the present study, we report on concordance between ELISA readouts and *in vivo* immunogenicity in a guinea pig model as it relates to vaccine dosing levels and sensitivity to thermal stress. We found correlation between *in vitro* ELISA values and neutralizing antibody responses engendered after animal immunization. Furthermore, this *in vitro* assay could be used to demonstrate the effect of thermal stress on vaccine potency, and such results could be correlated with physicochemical analysis of the recombinant protein antigens. This work demonstrates the suitability of the *in vitro* ELISA to measure vaccine potency and the correlation of these measurements to an immunologic outcome.

## 1 Introduction

Rotavirus is the leading cause of diarrhea-associated disease in children under 5 years of age, with a disproportionate amount of disease in low- and middle-income country (LMIC) settings [1], [2], [3]. Even with multiple licensed oral rotavirus vaccines on the market, the relatively high cost and reduced efficacy observed in LMIC settings (∼50 %) compared to high-income settings (>90 %) [4], [5] has limited their impact on rotavirus-associated disease [6], [7], [8], [9]. Several factors have been suggested to explain the reduced vaccine efficacy, including variations in gut microbiota and host mucosal factors, which may interfere with the resulting immune response [10]. Parenterally administered rotavirus vaccines [11], both subunit and inactivated whole virion, which may improve efficacy, are being developed to provide an alternate vaccine approach for LMIC settings [4], [12]. One such candidate, non-replicating rotavirus vaccine (NRRV), is composed of three truncated VP8 subunits of rotavirus (VP8 P[4], VP8 P[6], and VP8 P[8]) coupled to a universal tetanus toxoid CD4 + T cell epitope (*P*2) [13] that are adsorbed to an aluminum adjuvant (trivalent *P*2-VP8). After having been shown to be safe and immunogenic in 6–10-week-old infants [14], the efficacy of this candidate NRRV is currently being evaluated in a pivotal efficacy trial [15], [16], [17].

Multiple animal models, including mice, gnotobiotic piglets, neonatal rats, rhesus macaques, and guinea pigs, have been used to study rotavirus [18]. Each of these models has advantages and disadvantages, including susceptibility to disease versus infection, genetic differences, limited understanding of cellular responses, and effect of microbiota on rotavirus infection. In addition to the limitations of existing animal models, human immune correlates of protection from rotavirus are not well defined [18], presenting a particular challenge for non-replicating vaccine candidates, which will not elicit an immunoglobin A response as observed for oral rotavirus vaccines. Guinea pigs have been used throughout the development of the NRRV *P*2-VP8 candidate and were selected for the current studies based on prior experience with this vaccine candidate. Additionally, due to the uncertainty regarding the appropriate correlate of protection, both neutralizing antibody and total binding IgG levels were assessed in the current studies.

As part of the trivalent *P*2-VP8 NRRV development program, a battery of analytical tests, including antigen–antibody binding ELISAs, were developed to characterize the key structural attributes of each of the three antigens in this multivalent vaccine candidate. These methods were developed to help determine the influence of critical parameters on the vaccine’s potency and stability [19], [20]. In the current study, we investigated the concordance between *in vitro* ELISA readouts and results from a guinea pig immunogenicity model.

## 2 Materials and methods

Monovalent *P*2-VP8 bulk antigens (P[4], P[6], and P[8]) were kindly provided by SK bioscience (Seongnam, South Korea). Prior to use, we thawed monovalent antigens by holding at 2 °C-8 °C overnight and sterile-filtered (0.22 µm). Protein concentrations for each monovalent were determined by absorbance at 280 nm using antigen-specific extinction coefficients [21]. Adjuvanted monovalent and trivalent antigen formulations were prepared by adsorption to Alhydrogel® (Brenntag Nordic A/S, Denmark), an aluminum adjuvant in gel-like aqueous suspension (Al(OH)_3_) [20]. Adsorption to aluminum adjuvant for both monovalent and trivalent formulations was confirmed by measurement of the amount of protein remaining in the supernatant after adsorption. All formulations tested satisfied > 90 % of total protein adsorbed this exceeds the minimum vaccine release specification of > 70 % of total protein.

### 2.1 Dose-ranging study

Monovalent *P*2-VP8 antigens were formulated with Alhydrogel and combined into a trivalent vaccine formulation by the established methods [20], but at twice the final concentration (0.12 mg/mL of each antigen for a final trivalent concentration of 0.36 mg/mL total protein with 2.24 mg/mL Al in 0.5 mM phosphate buffered saline [PBS], pH 7) to allow for the guinea pig final dose (0.25 mL injection volume) to align with the human dose (0.5 mL injection volume) [20]. The resulting 2X trivalent adsorbed vaccine was diluted with 2.24 mg Al/mL Alhydrogel to achieve the respective target doses for immunization studies in the guinea pig dose-ranging study (30, 10, 3, 1, and 0.1 µg of each antigen per dose). Quantitation of aluminum-adsorbed *P*2-VP8 antigen was performed using the trivalent *P*2-VP8 inhibition ELISA and bicinchoninic acid (BCA) protein assay (Thermo Fisher Scientific™, #23225) methods as previously described [19].

### 2.2 Thermal stress vaccine preparation

Monovalent *P*2-VP8 vaccine formulations to be subjected to thermal stress were prepared by diluting *P*2-VP8 bulk antigens with 0.5 mM PBS to 0.72 mg/mL and mixing with 2.24 mg Al/mL Alhydrogel at an equal volume ratio to achieve a final concentration of 0.36 mg/mL monovalent adsorbed *P*2-VP8 vaccine with 1.12 mg Al/mL. Monovalent vaccine formulations were stored in 5-mL aliquots in sterile glass vials [20] placed upright and undisturbed at elevated temperature (thermally stressed vaccine) or at 2 °C-8 °C (control vaccine). P[4] and P[6] monovalent vaccine formulations were thermally stressed at 30 °C, and P[8] monovalent vaccine formulations were thermally stressed at 40 °C. Reduced antigenicity of thermally stressed monovalent *P*2-VP8 vaccine formulations was followed throughout the stability study using the *P*2-VP8 inhibition ELISA methods as previously described [19]. P[4] and P[6] monovalent vaccine formulations were tested on days 0, 3, 6, 7, 10, and 15, and P[8] monovalent vaccine formulations were tested on days 0, 6, 11, 13, 20, and 27. To prevent contamination and microbial growth, single-use vials were tested at each time point. Completion of the thermal stress was effectuated by an antigen content 50 % or less than the initial antigen concentration, as measured by the inhibition ELISA. Each of the monovalent antigens showed a different rate of loss of antigenicity by ELISA. Upon completion of the thermal stress, monovalent *P*2-VP8 vaccine formulations were transferred to 2 °C-8 °C until further use. Stressed monovalent *P*2-VP8 vaccines were allocated for either *in vitro* biochemical characterization or *in vivo* immunogenicity studies performed in guinea pigs. Monovalent *P*2-VP8 vaccines allocated for *in vivo* immunogenicity studies were co-formulated into a trivalent NRRV by equal volume mixing prior to dilution to the target dose concentrations (see below).

A trivalent NRRV formulation to be subjected to thermal stress was prepared by first diluting monovalent *P*2-VP8 bulk antigens (P[4], P[6], and P[8]) to 0.36 mg/mL with the sterile 0.5 mM PBS and then combining the antigens in equal volume ratios such that the total protein concentration was 0.36 mg/mL, and the concentration of each antigen was 0.12 mg/mL. The trivalent mixture of *P*2-VP8 antigens was then adsorbed to aluminum adjuvant by mixing with 2.24 mg Al/mL Alhydrogel at an equal volume ratio to achieve a final concentration of 0.18 mg/mL total protein (0.06 mg/mL of each antigen) and 1.12 mg Al/mL. The trivalent NRRV was stored in 5-mL aliquots in sterile glass vials and placed upright and undisturbed at 25 °C (thermally stressed vaccine) or at 2 °C-8 °C (control vaccine). Reduced antigenicity of each of the *P*2-VP8 antigens in the trivalent NRRV formulation was monitored by measuring the antigen concentration via their respective inhibition ELISA on days 0, 14, 21, 42, and 45. To prevent contamination and microbial growth, single-use vials were removed and tested at each time point. Completion of the thermal stress of trivalent NRRV was effectuated when the concentration of one of the antigens was 50 % or less than the initial antigen concentration, as measured by the trivalent inhibition ELISA [19]. Thermal stress of trivalent NRRV was completed on day 45, and the trivalent NRRV was transferred to 2 °C-8 °C until use in *in vivo* immunogenicity studies.

Upon completion of thermal stress treatment, the stressed trivalent NRRV and the trivalent combining the stressed monovalent *P*2-VP8 vaccines were diluted with 2.24 mg Al/mL Alhydrogel to achieve the following concentrations for use in guinea pigs: high-dose group, 36 µg/mL total protein (3 µg/mL of each antigen); medium-dose group, 12 µg/mL total protein (1 µg/mL of each antigen); and low-dose group, 1.2 µg/mL total protein (0.1 µg/mL of each antigen).

### 2.3 Biophysical characterization assays

Monovalent *P*2-VP8 aluminum adjuvanted vaccine formulations were evaluated by physiochemical methods in addition to *in vivo* testing. Experimental details of the physicochemical methods used in this work have been in general described previously [21], [22], [23], and the specific experimental setups and analytical methods for differential scanning calorimetry (DSC) and for “mild forced” and “strong forced” desorption treatments (to desorb the NRRV antigen from the aluminum adjuvant) are as follows:

### 2.4 Differential scanning calorimetry (DSC)

DSC analysis was performed using MicroCal VP-Capillary calorimeter (Malvern, United Kingdom) equipped with tantalum sample and reference cells. Samples were loaded into a temperature-controlled auto sampler tray held at 4 °C. Scans were completed from 10 °C to 90 °C using a scanning rate of 60 °C/h. Data analysis was performed using the DSC plug-in for the Origin 7.0 software package. After performing reference subtraction and concentration normalization, results were fitted to a “non-two-state” model with one transition to calculate the melting temperature (T_m_) value. The area under the curve (apparent enthalpy, ΔH’) was calculated using the peak integration function in the Origin 9.4 software package.

### 2.5 “Mild forced” desorption treatment to remove NRRV antigen from aluminum adjuvant followed by UV–visible spectroscopy analysis

Antigen-adjuvant sample (0.18 mg/mL antigen, 1.125 mg/mL aluminum) was centrifuged at 4,000 X g for 5 min to pellet the adsorbed antigen and adjuvant. Then 900 µL of the supernatant was removed, and the pellet was re-suspended in a mixture of 50 µL of 1 M sodium phosphate, pH 7.0 (0.2 M final concentration) and 100 µL of water (0.2 M sodium phosphate final concentration). The resuspended pellet was incubated at room temperature for 15 min in the dark, followed by centrifugation at 4,000 X g for 5 min. The UV–visible absorption spectra of the supernatant fraction of desorbed monovalent NRRV samples were recorded with an Agilent-8453 UV–visible spectrophotometer equipped with deuterium (D_2_) and tungsten (W) lamps. The Beer-Lambert law was used to calculate protein concentration based on calculated extinction coefficient 1.733 mg/mL^-1^cm^−1^ for P[8], 1.708 (mg/mL)- 1 cm-1 for P[6], and 1.653 mg/mL^-1^cm^−1^ for P[4] (calculated using protein sequence using https://web.expasy.org/protparam/). All UV-spectra were blank subtracted using a placebo sample (1.125 mg/mL Al in 0.5 mM PBS subjected to mild forced desorption) and corrected for light scattering using a technique included in the manufacturer’s data analysis software (ChemStation UV–vis analysis software; Agilent Technologies).

### 2.6 “Strong forced” desorption treatment to remove NRRV antigen from aluminum adjuvant followed by SDS-PAGE analysis

Antigen-adjuvant sample (0.18 mg/mL antigen, 1.125 mg/mL aluminum) was centrifuged at 4,000 X g for 5 min to pellet the adsorbed antigen and adjuvant. Then 900 µL of the supernatant was removed, and the pellet was resuspended in a mixture of 0.2 M sodium phosphate + lithium dodecyl sulfate (LDS) buffer (Life Technologies) + 20 mM iodoacetamide (Thermo Fisher Scientific) and incubated in the dark for 15 min at room temperature. Then the samples were heated at 90 °C for 10 min, followed by centrifugation at 4,000 X g for 5 min. Supernatant was recovered and divided in two parts to prepare non-reduced and reduced samples for SDS-PAGE analysis. For the reduced samples, supernatant was mixed with 10 mM dithiothreitol (Thermo Fisher Scientific); for the non-reduced samples, an equal volume of ultra-pure water was added and then the samples were incubated at 37 °C for 10 min. Finally, the reduced/non-reduced samples were separated by SDS-PAGE using NuPAGE™ 4 to 12 % Bis-Tris (Life Technologies) gels and MES SDS running buffer (50 mM 2-[*N*-morpholino]ethanesulfonic acid; Life Technologies). A theoretically equivalent amount of protein was also loaded on the gel as in-solution control (i.e., protein that was never exposed to adjuvant). The purpose of running in-solution control was to quantify the percent desorption under the forced desorption condition of phosphate + LDS sample buffer + boiling at 90 °C for 10 min by comparing the band intensities between in-solution control and desorbed samples by ImageJ (National Institutes of Health, United States) analysis. Gels were run first for 10 min at 120 V followed by 50 min at 150 V. Protein bands were visualized by staining with Coomassie Blue R-250 (Teknova, Hollister, CA) for 1 h and were then destained with a mixture of 40 % methanol, 10 % acetic acid, and 50 % ultrapure water. Gels were digitized using an Alphaimager (ProteinSimple, Santa Clara, CA) gel imaging system.

### 2.7 *In vivo* immunogenicity

Immunization of guinea pigs with trivalent NRRV were performed at Noble Life Sciences, Inc. (Sykesville, Maryland), in compliance with the Animal Welfare Act (US Code of Federal Regulations), US Public Health Service Policy on Human Care and Use of Laboratory Animals, National Academy of Sciences *Guide for the Care and Use of Laboratory Animals*, and Association for Assessment and Accreditation of Laboratory Animal Care International [24], [25]. Groups of 30 animals (male Hartley guinea pigs, approximately 300 g) were immunized intramuscularly (0.25 mL) on days 0, 14, and 28. Serum samples were collected on days 0, 28, and 42, and stored at –80 °C prior to testing.

Neutralizing antibody titers against vaccine homologous strains DS-1 (P[4]), 1076 (P[6]), and Wa (P[8]) in day 42 serum were measured at Cincinnati Children’s Hospital Medical Center (Cincinnati, Ohio) as previously described [20], [26].

Antigen-specific binding IgG responses were measured as previously described [20]. Antibody responses were measured in serum samples from a dose-ranging study after the second dose (day 28) and third dose (day 42) of trivalent *P*2-VP8 vaccine in guinea pigs. Antigen-specific binding IgG responses were also measured after the second immunization (day 28) and third immunization (day 42) of the thermal stress study in guinea pigs.

### 2.8 Statistical analysis

Data from the *in vivo* immunogenicity studies were performed by individual operators without replication. Titers were transformed using log base 2 prior to all analyses. Log_2_ titers were used to measure within- and between- sample and day variances at days 28 and 42 for binding IgG titers. Neutralizing antibody titer data were only available for day 42, so only within- and between- sample analytics were produced. A mixed-model analysis of variance (ANOVA) was used to quantify these variances and measure differences between days and dosages. While an increased number of non-responders were observed in the lowest doses studied, no significant differences were observed between the median and mean of these groups, suggesting this has no effect on the conclusions drawn. A p-value of less than or equal to 0.05 was considered significant.

## 3 Results

To identify the appropriate dose for conducting *P*2-VP8 immunogenicity studies in guinea pigs, a dose-ranging study was conducted. Five doses of the trivalent NRRV were tested, which ranged from 30 µg (current clinical human dose) to 10, 3, 1, and 0.1 µg of each antigen (P[4], P[6], and P[8]) adsorbed to a constant 0.56 mg Al per dose. The antigen content in the 30, 10, and 3 µg doses was confirmed by an inhibition ELISA and BCA (Table 1). The antigen content in the 1-µg and 0.1-µg doses, however, was below the limit of detection for both assays.

**Table 1:** Antigen content in dose-ranging study formulations of aluminum adjuvanted trivalent NRRV as measured by inhibition ELISA and BCA. LD indicates that the samples were below the limit of detection for this assay. Each antigen (P[4], P[6], and P[8]) and each dose was adsorbed to a constant 0.56 mg Al per dose.

				**ELISA**			**BCA**
**Group**	**Total µg/mL**	**Dose/ antigen (µg)**	**Injection volume** **(mL)**				
				**P[4] µg/mL** **± SD** **(% expected)**	**P[6] µg/mL** **± SD** **(% expected)**	**P[8] µg/mL** **± SD** **(% expected)**	**Total** ***P*2-VP8** **(µg/mL)**
**1**	360	30	0.25	130 ± 7.5 (108 %)	122 ± 5.8 (102 %)	108 ± 6.2 (90 %)	401
**2**	120	10	0.25	42 ± 3.4 (105 %)	35 ± 1.1 (88 %)	40 ± 0.7 (100 %)	136
**3**	36	3	0.25	9 ± 0.8 (75 %)	14 ± 0.6 (117 %)	10 ± 0.5^a^ (83 %)	29
**4**	12	1	0.25	4 ± 0.5^a^ (100 %)	LD	LD	LD
**5**	1.2	0.1	0.25	LD	LD	LD	LD

Key: BCA, bicinchoninic acid; LD, limit of detection; SD, standard deviation.

a Not enough points to meet system suitability requirements.

**Fig. 1:**
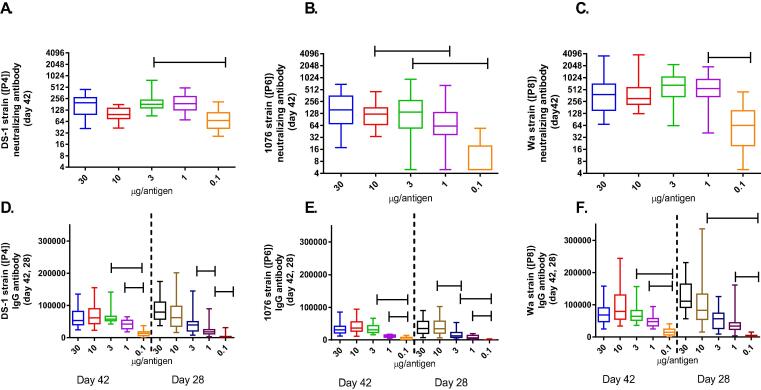
Immunogenicity results from trivalent NRRV dose-ranging study. Neutralizing antibodies geometric mean titers against rotavirus strains **A.** DS-1 (P[4]), **B.** 1076 (P[6]), and **C.** Wa (P[8]) were measured at day 42 after three immunizations (days 0, 14, and 28) of trivalent NRRV in guinea pigs over a dose range of 30–0.1 µg for each *P*2-VP8 antigen. Antigen-specific IgG geometric mean titer responses against **D.** DS-1P[4], **E.** 1076P[6], and **F.** Wa P[8] at day 28 after two immunizations and day 42 after three immunizations. Bars indicate statistically significant differences (p < 0.0001).

Serum samples from each animal were collected and analyzed for *P*2-VP8 specific neutralizing antibodies after the third immunization (Fig. 1, A–C, day 42). A statistically significant (p < 0.0001) decrease in neutralizing antibody response was observed for the lowest dose of the trivalent NRRV (0.1 µg per antigen) compared to the 1-µg dose for P[8] or the 3-µg dose for P[4] or P[6] antigens. In addition, the 1-µg trivalent *P*2-VP8 vaccine dose also showed a significantly decreased neutralizing antibody response compared to the 10-µg dose for the P[6] antigen but was not statistically different for the P[4] or P[8] antigens. *P*2-VP8 specific binding IgG responses were measured after the second (day 28) and third (day 42) immunization of trivalent NRRV in guinea pigs (Fig. 1, D–F). Trends in the binding IgG responses were similar to the neutralizing antibodies in which a significantly higher binding IgG response was measured in the 3- or 1-µg dose compared to the 0.1-µg dose. Based on these results, three doses (3, 1, and 0.1 µg of each *P*2-VP8 antigen) were selected for subsequent studies.

**Fig. 2:**
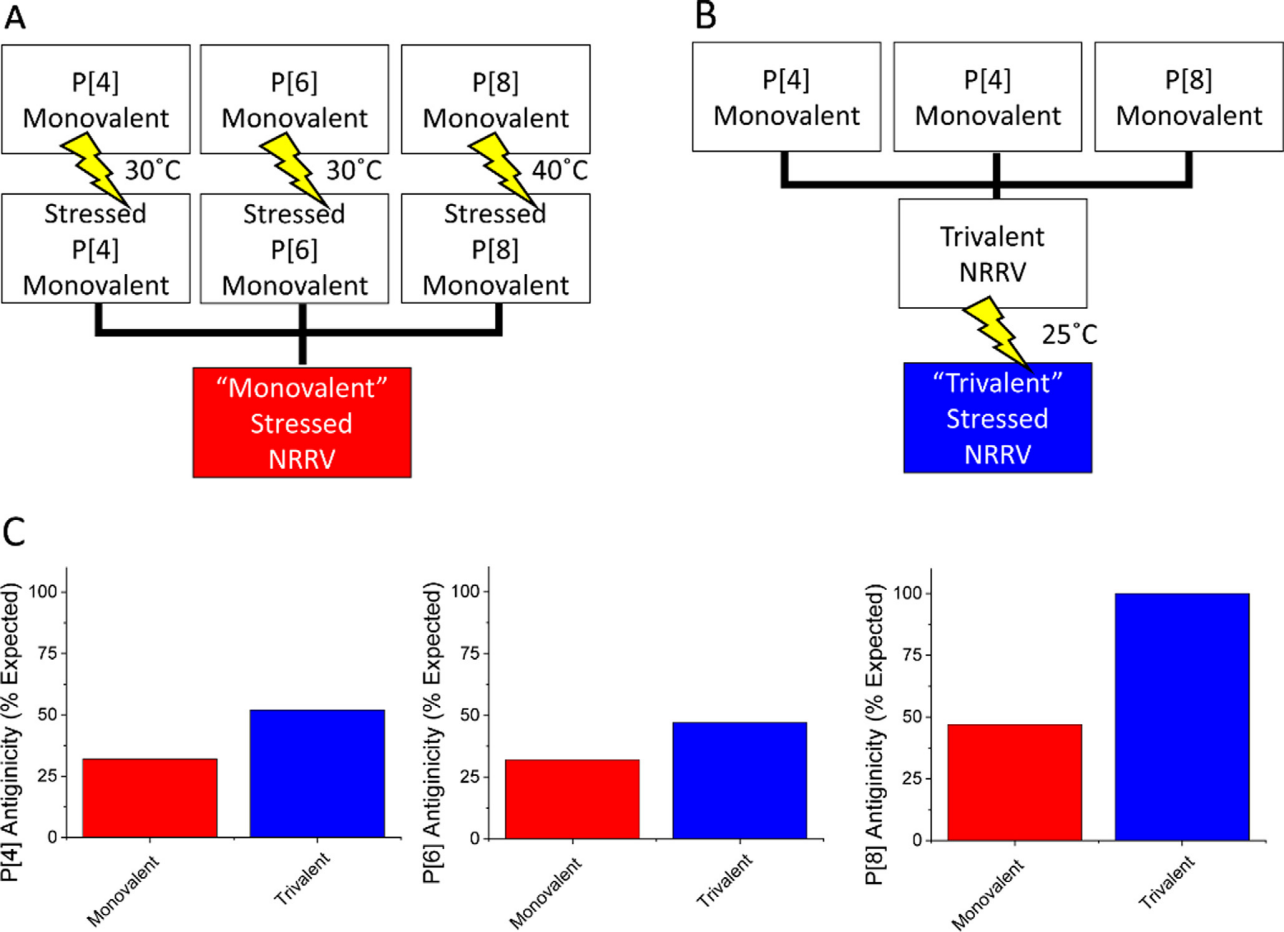
Schematic of the preparation of the stressed “monovalent” (**A**) and “trivalent” (**B**) aluminum adjuvant adsorbed NRRV formulations for animal testing. Yellow lightning bolts indicate application of thermal stress at the temperature indicated (**C**). Antigenicity of the *P*2-VP8 antigens (P[4], P[6], and P[8]) in the stressed monovalent and trivalent NRRV formulations by ELISA compared to an untreated trivalent control. Antigenicity was measured on a single replicate used in animal studies.

**Fig. 3:**
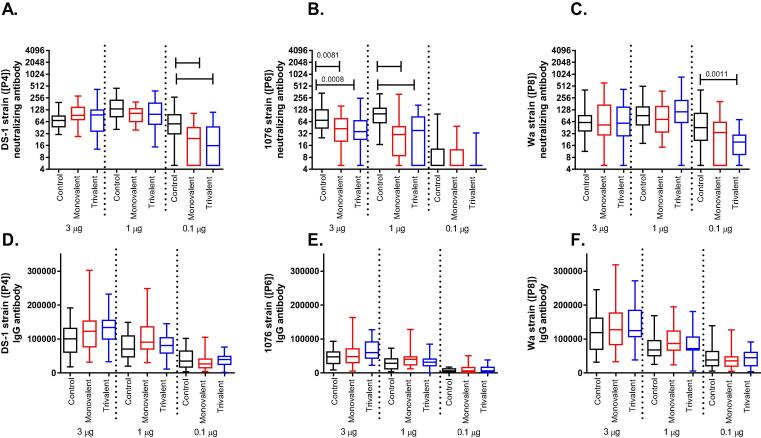
Immunogenicity results from thermal stress study of trivalent NRRV formulations. Neutralizing antibodies geometric mean titers against rotavirus strains **A.** DS-1 (P[4]), **B.** 1076 (P[6]), and **C.** Wa (P[8]) were measured at day 42 after three immunizations (days 0, 14, and 28) of trivalent NRRV in guinea pigs. Antigen-specific IgG geometric mean titer responses against **D.** DS-1P[4], **E.** 1076P[6], and **F.** Wa P[8] at day 42 after three immunizations. Bars indicate statistically significant differences with a p-value < 0.0001 unless otherwise noted.

**Fig. 4:**
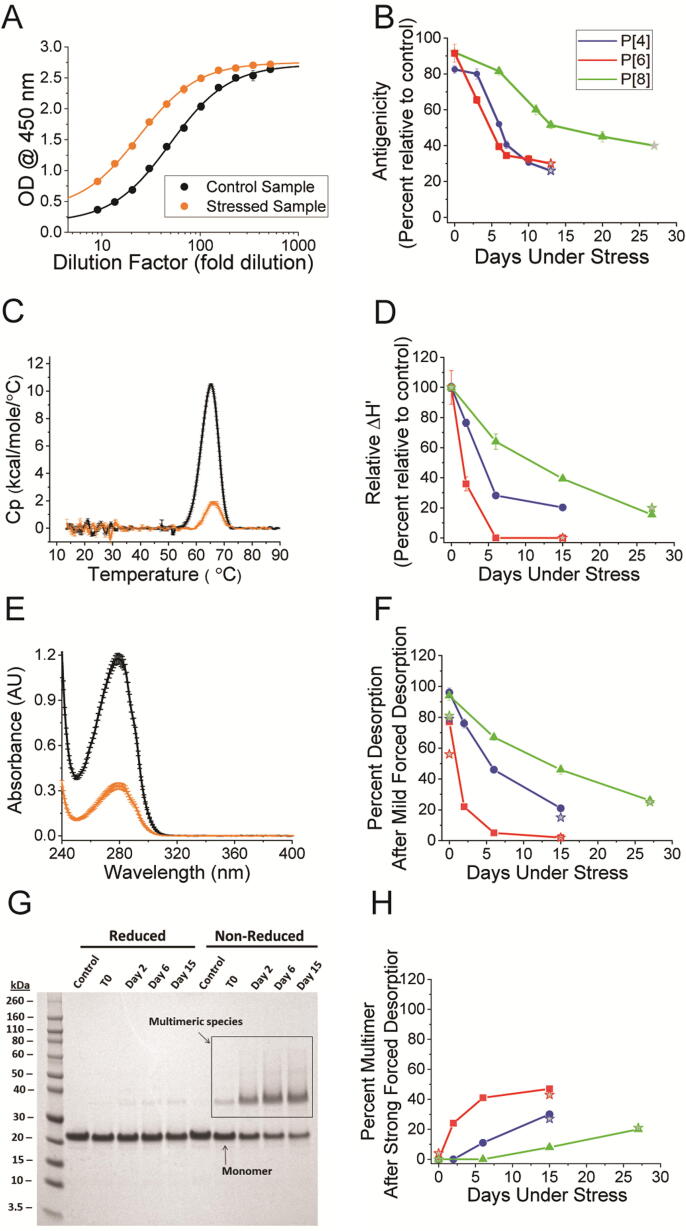
Thermal degradation profile of the three monovalent, aluminum-adjuvanted NRRV antigens (*P*2-VP8: P[8], P[6], P[4]). Representative measurements (left panels) of control (black) and stressed (orange) monovalent *P*2-VP8 P[8] (**A, C** and **E**) or *P*2-VP8 P[6] (**G**) and time-dependent degradation during storage at 30˚C (P[4] and P[6]) or 40˚C (P[8]) (right panels) of monovalent *P*2-VP8 P[4] (blue), P[6] (red), and P[8] (green) as measured by the *in vitro* ELISA antigenicity assay (**A** and **B**), apparent enthalpy (ΔH’) (**C** and **D**) as measured by DSC, percent antigen desorption following mild forced desorption (**E** and **F**) as measured by UV–visible spectroscopy, and the formation of multimers measured after strong forced desorption (G and H) as measured by SDS-PAGE. ≥ 90 % of total expected amount protein antigen was recovered following strong forced desorption treatment (data not shown).” Panel B: error bars represent standard deviation of duplicate measurements; singlet endpoint measurements are represented by filled stars. Panels **D**, **F**, and **H** display the cumulative results obtained from two distinct sample sets. The values obtained from triplicate measurements at four time points are represented as lines with data points. Singlet endpoint measurements are represented by filled stars.

Two formulations of thermally stressed trivalent NRRV were produced for use to compare *in vitro* antigenicity studies to *in vivo* immunogenicity studies. The first was produced by individually stressing monovalent *P*2-VP8 vaccine formulations prior to mixing into a trivalent formulation. The second was produced by mixing the three *P*2-VP8 antigens into a trivalent NRRV formulation prior to thermal stressing. The former is referred to as the “monovalent” and the latter is referred to as the “trivalent” to reflect the conditions in which they were thermally stressed (Fig. 2). Monovalent and trivalent vaccine formulations were thermally stressed targeting approximately a 50 % reduction in antigenicity as measured by the inhibition ELISA. Thermal stress conditions for each antigen were selected based on a preliminary accelerated stability study of trivalent vaccine that demonstrated different rates of change for each antigen in the formulation (data not shown). A stress condition of 30 °C was selected for monovalent P[4] and P[6] antigens, while 40 °C was selected for the monovalent P[8] antigen. For the trivalent NRRV, 25 °C was selected with the goal of achieving a decrease in antigenicity of at least 50 % for at least one of the antigens in the trivalent formulation. The antigenicity of the *P*2-VP8 antigens in the stressed monovalent and trivalent NRRV was measured by the *in vitro* ELISA assay (Fig. 3). Thermal stress of the monovalent P[4] and P[6] sample was completed on day 13, at which point the remaining antigenicity was ∼ 30 % of the expected for each antigen. Thermal stress of monovalent P[8] sample was completed on day 27, at which point the remaining antigenicity was ∼ 40 % of the expected. Thermal stress of trivalent NRRV was completed on day 45, at which point the P[4] antigen content had decreased to 52 % of expected, the P[6] antigen content had decreased to 47 % of expected, and the P[8] antigen content remained unchanged, as measured by the trivalent inhibition ELISA (Fig. 2C). Due to material limitations, the terminal measurement of antigenicity was performed with a single replicate for each antigen in the monovalent and trivalent formulations. A full time-course of measurements performed during the thermal stress of monovalent and trivalent NRRV formulations is presented in Fig. 4B and Supplemental Figure S1, respectively.

The *in vivo* relevance of the loss in antigenicity was assessed by measuring the immunogenicity of the stressed monovalent and trivalent formulations in guinea pigs. NRRV neutralizing antibodies were evaluated after three doses (day 42) of either thermally stressed or non-stressed (control) vaccine formulations (Fig. 3, A–C). Statistically significant differences were observed for each rotavirus strain (P[4], P[6], and P[8]) for the doses selected. P[4] neutralizing antibody responses against the control vaccine were significantly different between the 3-µg dose and the 1-µg dose (p = 0.0008) as well as the 1–µg dose and the 0.1-µg dose (p < 0.0001). P[6] neutralizing antibody responses against the control vaccine were significantly different between the 3-µg dose and the 0.1-µg dose (p < 0.0001) as well as the 1-µg dose and the 0.1-µg dose (p < 0.0001). P[8] neutralizing antibody responses against the control vaccine showed modest differences between the 3-µg dose and the 1–µg dose (p = 0.0473) as well significant differences between the 1–µg dose and the 0.1–µg dose (p = 0.0004). The statistically significant differences observed between different dose levels tested in the control groups is consistent with the findings of the first dose-ranging study and confirms the dose range selected. Significant differences were also observed between control and thermally stressed vaccine formulations for each of the rotavirus strains. Significant differences were observed for P[4] neutralizing antibody responses between the control and thermally stressed vaccine (monovalent and trivalent) groups at the 0.1–µg dose level (p < 0.0001). Significant differences were observed for P[6] neutralizing antibody responses between the control and thermally stressed vaccine (monovalent and trivalent) groups at the 1– and 3–µg dose levels. Significant differences were observed for P[8] neutralizing antibody responses between the control and trivalent thermally stressed vaccine group at the 0.1–µg dose level (p = 0.0011). No significant differences were observed between the monovalent and trivalent thermally stressed groups for any of the rotavirus types. Total binding IgG antibodies were also evaluated after two immunizations on day 28 Supplementary Figure S2) and after three immunizations on day 42 (Fig. 3, D–F). Total binding IgG showed similar dose response trends to both the previous dose-ranging study as well as the neutralizing antibody results, although the differences were not statistically significant.

The degradation of thermally stressed monovalent *P*2-VP8 samples was followed by measuring the relative antigenicity at four time points at elevated temperature (30 °C for P[4] and P[6], 40 °C for P[8]) using the *in vitro* ELISA assay (see Fig. 4, A and B for representative data and time-dependent degradation, respectively). P[8] is seen as the most stable of the three monovalent *P*2-VP8 antigens, followed by P[4], which is seen as only slightly more stable that P[6] (Fig. 4B). In conjunction with the animal studies, the control and thermally stressed monovalent samples were characterized using a comprehensive suite of *in vitro* analytical methods to provide insight into the mechanism of the decreased neutralization response. Due to material and analytical limitations, physiochemical analysis was limited to one replicate of the monovalent *P*2-VP8 samples prior to formulating the trivalent NRRV used in the animal studies. Since substantial structural and chemical alterations were observed in the stressed samples, an expanded study was conducted to evaluate the physicochemical properties of the monovalent *P*2-VP8 antigens in triplicate and at four time points while exposed to elevated temperature. The results of both the single replicate of monovalent *P*2-VP8 samples used in the animal study as well as the subsequently performed expanded study are shown in Fig. 4, C—H (closed stars and lines graph with data points, respectively). The following biophysical properties of each *P*2-VP8 antigen were assessed during thermal stress: antigenicity (by ELISA), overall conformational stability (by DSC), changes to the interaction between each antigen and aluminum adjuvant (by UV–visible absorbance spectroscopy following “mild” forced desorption conditions and by SDS-PAGE following “strong” forced desorption conditions), and changes to the oligomeric state of each antigen (by reduced versus non-reduced SDS-PAGE). “Mild forced” desorption refers to experimental conditions that remove the NRRV antigens from the aluminum adjuvant yet are not expected to perturb the overall structural integrity of the protein (see methods). “Strong forced” desorption refers to experimental conditions that denatures the protein antigen (heat and addition of SDS) and completely removes (≥90 %) of the protein antigen from the aluminum adjuvant (see methods). When performed under both reduced vs non-reduced conditions, the “strong-forced” desorption also measures the presence of oligomeric protein due to formation of non-native disulfide bonds.

As expected, the antigenicity of each *P*2-VP8 antigen decreased following increasing exposure to elevated temperatures (Fig. 4, A and B). In addition, the relative apparent enthalpy (ΔH’) measured in each sample decreased over time (Fig. 4, C and D). Conversely, the ability to desorb each antigen from the aluminum adjuvant under mild forced conditions decreased (Fig. 4, E and F). Following strong forced desorption under reducing or non-reducing conditions, the percentage of *P*2-VP8 antigen in the multimer conformation increased with increased exposure to elevated temperature (Fig. 4, G and H). These results indicate that as the thermal stress induced conformational changes within each *P*2-VP8 antigen, it decreased epitope availability/integrity and increased the interactions with both the aluminum adjuvant and with neighboring *P*2-VP8 antigens.

## 4 Discussion

Rotavirus is the leading cause of diarrhea-associated disease in children. Although licensed rotavirus vaccines are available, development of additional vaccines is critical for addressing the large medical need. The nonreplicating trivalent *P*2-VP8 rotavirus vaccine (NRRV) currently in Phase 3 clinical trials is a promising option for combating rotavirus in LMICs [16], [27], [28]. Vaccines composed of well-defined antigens with appropriate tests for characterization obviate the need for extensive testing in animals for vaccine release and monitoring potency. Practically eliminating the need for animal testing is attractive because it saves time and budgets, is in line with the 3R principles (Replace, Reduce, Refine), and reduces the variability associated with animal-based tests. However, removing animal testing from routine vaccine testing requires multiple qualified assays, of which the ELISA is one [29], [30]. Part of trivalent *P*2-VP8 vaccine development includes understanding the correlation between *in vitro* and *in vivo* test methods. This study supports the correlation of *in vitro* ELISA for measuring *P*2-VP8 vaccine antigenicity with *in vivo* animal immunogenicity testing. The ELISA and the guinea pig model demonstrated the ability of both methods to discriminate between different dose levels of the vaccine as well as changes in the vaccine antigens due to thermal stress. These correlations were observed for each antigen present in the trivalent vaccine.

The NRRV dose used for immunization of infants in the ongoing Phase 3 clinical trials is 30 µg of each antigen (90 µg total antigen) and 0.5625 mg Al/mL Al(OH)_3_ [17]. This dose was selected based on Phase 2/3 clinical trials evaluating the safety and efficacy of the trivalent NRRV [14]. In infant immunizations, the 30-µg dose is achieved via a 0.5-mL intramuscular injection of trivalent NRRV containing 60 µg/mL of each antigen (180 µg/mL total antigen) with 1.125 mg Al/mL Alhydrogel. To achieve the same dose in the reduced injection volume used for intramuscular immunization of guinea pigs (0.25 mL vs 0.5 mL), a proportionally higher concentrated formulation of trivalent NRRV was used (360 µg/mL total antigen adsorbed to 2.25 mg Al/mL Alhydrogel). The quantitation of *P*2-VP8 antigens as measured by the *in vitro* antigenicity assay is accurate (+/- 25 %) from 360 to 36 µg/mL total antigen (120–12 µg/mL of each antigen) (Table 1). The immune response measured in guinea pigs was consistent across this range of concentrations (Fig. 1). This range of 360–36 µg/mL total antigen represents 200 % to 20 % of the in-use trivalent NRRV concentration. At 12 µg total antigen/mL, the trivalent NRRV is below the limit of detection of the *in vitro* antigenicity or antigen assay (Table 1), yet only a marginal decrease in the immunogenicity of P[6] and no decrease in the immunogenicity of P[4] or P[8] was observed relative to the 36 µg total antigen/mL sample (Fig. 1, 1-µg dose versus 3-µg dose). These results indicate that the *in vitro* antigenicity assay is more sensitive to conformational destabilization and/or material loss than the *in vivo* immunogenicity assay, which yields similar antibody titers despite a 30-fold reduction in antigen content. However, both *in vitro* and *in vivo* measures showed correlation for both decreased dose levels and loss of antigenicity due to stress.

The three dosages used in the *in vivo* immunogenicity study of stressed trivalent NRRV (3, 1, and 0.1 µg/mL) were down-selected from the dose–response study as the three doses most likely to yield a significant difference in antibody titers as a result of thermal degradation of the *P*2-VP8 antigens. A stressed trivalent NRRV formulation was produced by two different methods: either thermal stressing monovalent *P*2-VP8 vaccines prior to mixing into a trivalent vaccine or mixing monovalent *P*2-VP8 vaccines into a trivalent NRRV formulation prior to stressing. The value of these two different approaches is the formation of two different antigenicity profiles. The first approach (monovalent) produces a trivalent NRRV formulation degradation profile in which each antigen is approximately equally degraded by using a temperature and incubation time specific to the relative stability of each *P*2-VP8 antigen. The second approach more closely reflects a real-world stress condition; however, the resulting trivalent NRRV formulation had a disproportionate amount of degraded P[4] and P[6] relative to P[8]. By comparing the *in vitro* antigenicity to the *in vivo* immunogenicity using both stressed trivalent NRRV formulations, a balance between antigen-specific relevance and real-world relevance is achieved. Given that the dose–response study demonstrated that the 3-µg dose and 1-µg dose induced similar levels of P[4] and P[8] neutralizing antibodies, it is understandable that the 50 %–70 % reduction in antigenicity measured for the stressed trivalent NRRV formulations did not result in a reduction in immunogenicity for the P[4] and P[8] antigens in the 3-µg dose. The dose–response study indicated that 1 µg P[6] induced reduced neutralizing antibody titers relative to the 3-µg dose. In accordance, the ≥ 50 % decrease in P[6] antigenicity in the stressed monovalent and trivalent NRRV formulations resulted in a decrease in neutralizing antibody titers in the 3-µg dose (Fig. 3). The trend of reduced neutralizing antibody titers in the stressed monovalent and trivalent NRRV samples relative to the control NRRV samples continued in the 1 and 0.1 µg doses. The ≥ 50 % reduction in P[4] and P[8] antigenicity observed in the stressed monovalent NRRV formulation resulted in a reduction in the neutralizing antibody titers in the 0.1 µg/mL dose, demonstrating that the reduction in antigenicity measured by the *in vitro* ELISA was predictive of the immunogenicity in the *in vivo* guinea pig studies. However, the failure to observe a reduction in the neutralizing antibody titers in the 1-µg dose suggests that a trivalent NRRV formulation remains immunogenic when it contains approximately 0.5 µg of each antigen. This dose was not assessed in the dose–response curves. Potentially, this study could have been improved by measuring the immunogenicity study of stressed monovalent and trivalent NRRV at a dose between 1 and 0.1 µg.

Biophysical characterization of the thermally stressed, aluminum-adsorbed NRRV formulation was performed using *in vitro* ELISA, DSC, UV–visible spectroscopy following mild forced desorption, and SDS-PAGE following strong forced desorption. Biophysical characterization was limited to the monovalent *P*2-VP8 vaccine formulations to discern antigen-specific trends more efficiently without the need for deconvolution. DSC measurements of thermally stressed monovalent *P*2-VP8 vaccine formulations indicate a progressive reduction in the value of apparent enthalpy (ΔH’) values with increasing thermal stress. No change in the value of the thermal melting temperature (T_m_) was observed for the thermally stressed samples (Supplementary Table S1). A decrease in the value of ΔH’ without a concurrent change in the value of T_m_ likely indicates formation of a heterogeneous mixture of structurally altered species (adsorbed to the aluminum adjuvant surface) without a discrete T_m_. Following “mild forced” desorption treatment (see methods), initially ≥ 90 % of the adsorbed NRRV protein antigen was desorbed from the aluminum adjuvant. A reduction in the percent of protein antigen desorbed from aluminum adjuvant by mild forced condition during the stability study suggests conformational changes that increase the strength of antigen interaction with adjuvant. In contrast, following “strong forced” desorption treatment which denatures the protein followed by SDS-PAGE analysis (see methods), ≥ 90 % of the NRRV protein was desorbed from the aluminum adjuvant confirming the protein mass was still present. The “strong forced” desorption results also showed some percent of the NRRV antigens had formed oligomeric, disulfide crosslinked protein.

Thus, the physicochemical characterization of the thermally stressed *P*2-VP8 vaccine formulations was marked by a reduced desorption efficiency under mild forced desorption conditions and an increased percentage of multimers following strong forced desorption (Fig. 4). Together, the results of DSC, UV–visible spectroscopy, and SDS-PAGE suggest a generalized destabilization pathway of AH-bound NRRV antigen in which thermal stress structurally alters the *P*2-VP8 antigens, exposes residues in the hydrophobic core which results in both the formation of inter-protein disulfide bonds (i.e., multimers) as well as strengthening interactions with the Al(OH)_3_ adjuvant.

The decrease in the value of ΔH’ and the decrease in desorption efficiency under mild forced desorption conditions generally correlated with the decrease in antigenicity measured by the *in vitro* ELISA assay in regard to the order of relative stability (P[8] > P[4] > P[6]) and overall trend, but differs in the magnitude of change. Most notably, the relative value of ΔH’ and percent desorption of P[6] reaches ∼ 0 % by day 6, whereas the relative antigenicity levels off at about 50 %. This suggest that the destabilized form of the aluminum adsorbed monovalent *P*2-VP8 antigen likely maintains some affinity for the antibody used in the *in vitro* ELISA assay. Similar studies have been conducted with vaccines for hepatitis B, human papillomavirus, and respiratory syncytial virus to support transition from variable, costly, and time-consuming animal potency assays to *in vitro* methods. With hepatitis B, for example, Merck licensure was initially conducted using a mouse potency assay, but after licensure potency measures were transitioned to a commercial enzyme immunoassay, which proved to be more reliable and improved the accuracy of potency determinations [29], [31], [32].

The *P*2-VP8 ELISA allows for quantification of each antigen present in the trivalent vaccine in the presence of aluminum adjuvant [19]. These methods are considered measures of antigenicity and not traditional potency assays because the antigen-specific monoclonal antibodies do not recognize a neutralizing epitope, which allows for correlation to a relevant immunologic endpoint. The loss of structural integrity induced by thermal stress was measurable by the *P*2-VP8 ELISA as a reduction in antigenicity. Additionally, both the neutralizing antibody responses and the total specific IgG responses measured in the *in vivo* immunogenicity studies correlated with the *in vitro* inhibition ELISA antigenicity results. While there is no established correlate of protection for oral rotavirus vaccines let alone a novel parenteral vaccine such as trivalent *P*2-VP8, the correlation between *in vivo* immunogenicity and *in vitro* antigenicity supports the applicability of the *in vitro* antigenicity methods for monitoring vaccine quantity and stability [33]. This work supports the use of this *in vitro* method to evaluate antigen content in the final vaccine product and demonstrated correlation of this measure with immunologic outcomes in place of *in vivo* animal testing for vaccine potency [29].

## 5 Authors’ contributions

DM, ME, JS, and JAW completed *in vitro* ELISA and animal work.

DH, NS, JMH, PK, and JAW completed the *in vitro* physiochemical work.

DM, DH, ME, JS, and JAW drafted the manuscript.

SJ, DV, RS, and SC reviewed data and provided technical guidance in addition to reviewing the manuscript.

BP completed statistical analysis.

All authors attest they meet the ICMJE criteria for authorship.

## Declaration of Competing Interest

The authors declare that they have no known competing financial interests or personal relationships that could have appeared to influence the work reported in this paper.
