# Supplementary material for: Concordance of in vitro and in vivo measures of non-replicating rotavirus vaccine potency
Source: Vaccine. 2022 Aug 12;40(34):5069–78. doi: 10.1016/j.vaccine.2022.07.017 (PMC9405915; doi:10.1016/j.vaccine.2022.07.017)
Supplement: Supplementary data 1 [file mmc1.docx]

**Supplement**


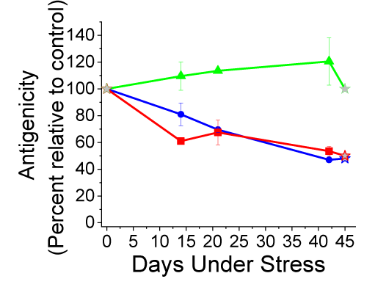


**Supplementary Figure S1.** The relative antigenicity stability profile of P2-VP8 P[4] (blue), P[6] (red), and P[8] (green) in the aluminum adjuvant adsorbed, trivalent NRRV samples over time as measured by the *in vitro* ELISA antigenicity assay. Trivalent NRRV was stressed at 25°C for up to 45 days as described in the Materials and Methods section. Errors bars reflect the standard deviation of n = 2 replicates; singlet endpoint measurements are represented by filled stars.

**Supplementary Figure S2.** P2-VP8 binding IgG at day 28 in a guinea pig thermal stress study. Guinea pigs were immunized with trivalent NRRV that was subjected to thermal stress either as a monovalent formulation or as a trivalent formulation prior to immunization. Control groups were immunized with untreated trivalent vaccine held at 2°C–8°C prior to use. Neutralizing antibodies were measured 14 days after the second immunization on day 28. **A.** Neutralizing antibodies against rotavirus strain DS-1 (P[4]). **B.** Neutralizing antibodies against rotavirus strain 1076 (P[6]). **C.** Neutralizing antibodies against rotavirus strain Wa (P[8]). Geometric mean titers at day 28 are shown.

|  | **Thermal melting temperature, Tm (°C)** | | |
| --- | --- | --- | --- |
| **Days under stress** | **NRRV antigen** | | |
|  | **P[4]** | **P[6]** | **P[8]** |
| 0 | 56.7 ± 0.1 | 56.4 ± 0.1 | 65.4 ± 0.1 |
| 2 | 57.0 ± 0.2 | 57.0 ± 0.1 | N/A |
| 6 | 57.0 ± 0.6 | ≤ LOD | 65.5 ± 0.2 |
| 15 | 56.0 ± 0.4 | ≤ LOD | 65.9 ± 0.2 |
| 27 | N/A | N/A | 65.6 ± 0.1 |

**Supplementary Table S1.** Thermal melting temperature (T­_m_) values as measured by DSC for P[4], P[6] and P[8] monovalent NRRV vaccine formulations after the indicated number of days of storage at either 30˚C (P[4] and P[6]) or 40˚C (P[8]). Values are reported as the average and one standard deviation of triplicate measurements. N/A: no value was measured for P[8] on day 2, or for P[4] or P[6] on day 27. No T_m_ values were determinable for P[6] after 6 or more days of incubation at 30˚C due to the value of ΔH’ decreasing below the limit of detection (LOD).
